# Supplementary material for: Investigating the Role of Friendship Interventions on the Mental Health Outcomes of Adolescents: A Scoping Review of Range and a Systematic Review of Effectiveness
Source: Int J Environ Res Public Health. 2023 Jan 25;20(3):2160. doi: 10.3390/ijerph20032160 (PMC9915149; doi:10.3390/ijerph20032160)
Supplement: Supplementary file 1 [file ijerph-20-02160-s001.zip › ijerph-2136700-supplementary.pdf]

Supplementary Materials S1

Sample search criteria

Web of Science
(minor* OR boy* OR girl* OR school* OR pupil* OR adolescen* OR juvenil* OR youth* OR teen* OR pubescen* OR prepub* OR pre-pub* OR preadolescen* OR pre-adolescen* OR "young adult*" OR "young people" OR "young person" OR "young man" OR "young men" OR "young woman" OR "young women" OR student*) (Topic) and (friend* OR peer* OR "authentic social group*") (Topic) and (depress* OR anxiety OR anxious OR "mental health" OR suicid* OR "eating disorder*" OR bulimi* OR anorexi* OR anhedonia OR wellbeing* OR well-being) (Topic) and (intervention* OR ipt OR "interpersonal psychotherapy" OR "study" OR "trial) (Topic) and 2022 or 2021 or 2020 or 2019 or 2018 or 2017 or 2016 or 2015 or 2014 or 2013 or 2012 or 2011 or 2010 or 2009 or 2008 or 2007 or 2006 or 2005 or 2004 or 2003 or 2002 or 2001 or 2000 (Publication Years)

ERIC
TX ((minor* OR boy* OR girl* OR school* OR pupil* OR adolescen* OR juvenil* OR youth* OR teen* OR pubescen* OR prepub* OR pre-pub* OR preadolescen* OR pre- adolescen* OR "young adult*" OR "young people" OR "young person" OR "young man" OR "young men" OR "young woman" OR "young women" OR student*)) AND TX ((friend* OR peer* OR "authentic social group*")) AND TX ((depress* OR anxiety OR anxious OR "mental health" OR suicid* OR "eating disorder*" OR bulimi* OR anorexi* OR anhedonia OR wellbeing* OR well-being)) AND TX ((intervention* OR ipt OR "interpersonal psychotherapy" OR study OR trial))
Limiters - Date Published: 20000101-20211231 Expanders - Apply equivalent subjects
Search modes - Boolean/Phrase
